# Predictors of the risk of malnutrition among children under the age of 5 years in Somalia

**DOI:** 10.1017/S1368980015001913

**Published:** 2015-06-16

**Authors:** Damaris K Kinyoki, James A Berkley, Grainne M Moloney, Ngianga-Bakwin Kandala, Abdisalan M Noor

**Affiliations:** 1 Department Public Health Research, Spatial Health Metris Group, INFORM Project, Kenya Medical Research Institute/Wellcome Trust Research Programme, PO Box 43640-00100, Nairobi, Kenya; 2 Kenya Medical Research Institute/Wellcome Trust Research Programme, Centre for Geographic Medicine Research (coast), Kilifi, Kenya; 3 Centre for Clinical Vaccinology and Tropical Medicine, Nuffield Department of Medicine, University of Oxford, Churchill Hospital, Oxford, UK; 4 Nutrition Section, UNICEF, Kenya Country Office, UN Complex Gigiri, Nairobi, Kenya; 5 Warwick Medical School, Health Sciences Research Institute, University of Warwick, Coventry, UK; 6 Division of Epidemiology and Biostatistics, School of Public Health, University of Witwatersrand, Johannesburg, South Africa; 7 Centre for Tropical Medicine and Global Health, Nuffield Department of Clinical Medicine, University of Oxford, Oxford, UK

**Keywords:** Malnutrition, Wasting, Stunting, Mid-upper arm circumference, Somalia

## Abstract

**Objective:**

To investigate the predictors of wasting, stunting and low mid-upper arm circumference among children aged 6–59 months in Somalia using data from household cross-sectional surveys from 2007 to 2010 in order to help inform better targeting of nutritional interventions.

**Design:**

Cross-sectional nutritional assessment surveys using structured interviews were conducted among communities in Somalia each year from 2007 to 2010. A two-stage cluster sampling methodology was used to select children aged 6–59 months from households across three livelihood zones (pastoral, agro-pastoral and riverine). Predictors of three anthropometric measures, weight-for-height (wasting), height-for-age (stunting) and mid-upper arm circumference, were analysed using Bayesian binomial regression, controlling for both spatial and temporal dependence in the data.

**Setting:**

The study was conducted in randomly sampled villages, representative of three livelihood zones in Somalia.

**Subjects:**

Children between the ages of 6 and 59 months in Somalia.

**Results:**

The estimated national prevalence of wasting, stunting and low mid-upper arm circumference in children aged 6–59 months was 21 %, 31 % and 36 %, respectively. Although fever, diarrhoea, sex and age of the child, household size and access to foods were significant predictors of malnutrition, the strongest association was observed between all three indicators of malnutrition and the enhanced vegetation index. A 1-unit increase in enhanced vegetation index was associated with a 38 %, 49 % and 59 % reduction in wasting, stunting and low mid-upper arm circumference, respectively.

**Conclusions:**

Infection and climatic variations are likely to be key drivers of malnutrition in Somalia. Better health data and close monitoring and forecasting of droughts may provide valuable information for nutritional intervention planning in Somalia.

Malnutrition is a leading cause of childhood deaths in low- and middle-income countries^(^ ^1^ ^)^ and has permanent consequences for cognitive, physical and metabolic development^(^ ^2^ ^)^. The burden of acute malnutrition is often greatest in areas suffering complex emergencies such as drought or conflict^(^ ^3^ ^)^. Somalia has been without a nationally recognized central government since 1991 and is one of the most unsafe countries globally^(^ ^4^ ^)^. This long period of insecurity and political instability has affected all facets of human life and development^(^ ^5^ ^)^. Somalia is now ranked the fifth poorest country globally and has among the highest child and maternal mortality rates^(^ ^6^ ^)^. It is also ranked lowest on the food security index^(^ ^6^ ^)^ and currently is estimated to have the highest rate of acute malnutrition in the world^(^ ^4^ ^)^. In 2011, approximately 2·9 million Somalians, or 35 % of the population, experienced food crisis^(^ ^7^ ^)^.

In the last two decades, international and local non-governmental organizations have supported essential public health services in Somalia. Primary health-care services are funded by several international organizations and donors, coordinated under the umbrella of the Somalia Support Secretariat, which was established under the auspices of a network including donors, UN agencies and international non-governmental organizations^(^ ^4^ ^)^. Interventions are often implemented under highly insecure conditions, especially in the southern parts of the country^(^ ^8^ ^)^. Interventions against food insecurity and malnutrition have been a major focus, including out-patient therapeutic feeding programmes for severe acute malnutrition and targeted supplementary feeding programmes for moderately malnourished children under the age of 5 years and pregnant or lactating women^(^ ^4^ ^)^.

In 1994 the World Food Programme set up the Food Security Analysis Unit, later renamed the Food Security and Nutritional Analysis Unit (FSNAU), to provide timely information to monitor and inform interventions to mitigate food insecurity and malnutrition in Somalia^(^ ^4^ ^,^ ^9^ ^)^. Since 2011, the FSNAU has undertaken several random cluster nutritional surveys including demographic, household and anthropometric data. These surveys have been used to inform the nature and timing of nutritional interventions across the country and provide alerts for acute food insecurity. Data from these surveys provide an opportunity to identify the determinants of childhood malnutrition during the complex emergency situation in Somalia.

Although inadequate nutritional intake is an obvious direct cause of malnutrition, many other factors are involved in causation, including poverty and access to health care, water and sanitation. These may be potential targets for interventions that could have a more sustained effect than reactive nutritional programmes^(^ ^10^ ^–^ ^14^ ^)^. Unravelling the determinants that are most important in this specific context can inform the efficient targeting of such interventions.

The determinants of childhood nutritional status can be classified as either proximate or distal^(^ ^15^ ^)^. Proximate factors have been the focus of most published literature and are child specific, including age, sex, inadequate nutritional intake and infections^(^ ^2^ ^,^ ^16^ ^,^ ^17^ ^)^. Distal factors include features of the wider socio-cultural, economic, environmental, climatic and political context that affect household food security, personal security and access to economic opportunities, health care and education^(^ ^18^ ^)^. These factors are likely to vary spatially and temporally, depending on geographic and climatic conditions and anthropogenic factors, and analyses must account for these variations.

In the present study, the first nationwide investigation of the predictors of malnutrition among children aged 6–59 months in Somalia is undertaken using the data from the FSNAU household cross-sectional nutritional surveys done from 2007 to 2010. Bayesian spatial-temporal regression models are applied to the data to account for the space and time effects on wasting, stunting and mid-upper arm circumference (MUAC).

## Methods

### Nutritional interventions context

In Somalia, a nutrition intervention group comprised of UNICEF, the World Food Programme and other government and non-governmental agencies was formed in 2006 to strengthen coordination of efforts against malnutrition^(^ ^6^ ^,^ ^19^ ^)^. This group developed a nutrition strategy for the period 2011–2013 in response to persistently high rates of malnutrition in the country. So far, several nutrition initiatives have been rolled out including the out-patient therapeutic feeding programmes for the management of severe acute malnutrition implemented by UNICEF and other agencies and the targeted supplementary feeding programmes for the management of moderately malnourished under-5s and pregnant and lactating women supported by the World Food Programme^(^ ^19^ ^)^. UNICEF currently targets 100 000 children aged 6–36 months with blanket distribution of ready-to-use food every two months in areas showing the highest malnutrition rates. The World Food Programme is also providing food assistance to vulnerable groups through institutional feeding and school feeding to about 90 000 beneficiaries. General food rations, consisting of cereals, corn–soya blend, sugar, fortified oil and iodized salt when available, are distributed to vulnerable rural populations, the urban poor and internally displaced persons. Despite these efforts, these interventions are thought to cover only a small proportion of the children under the age of 5 years who are likely to be malnourished in Somalia.

### Nutritional survey data

**Fig. 1:**
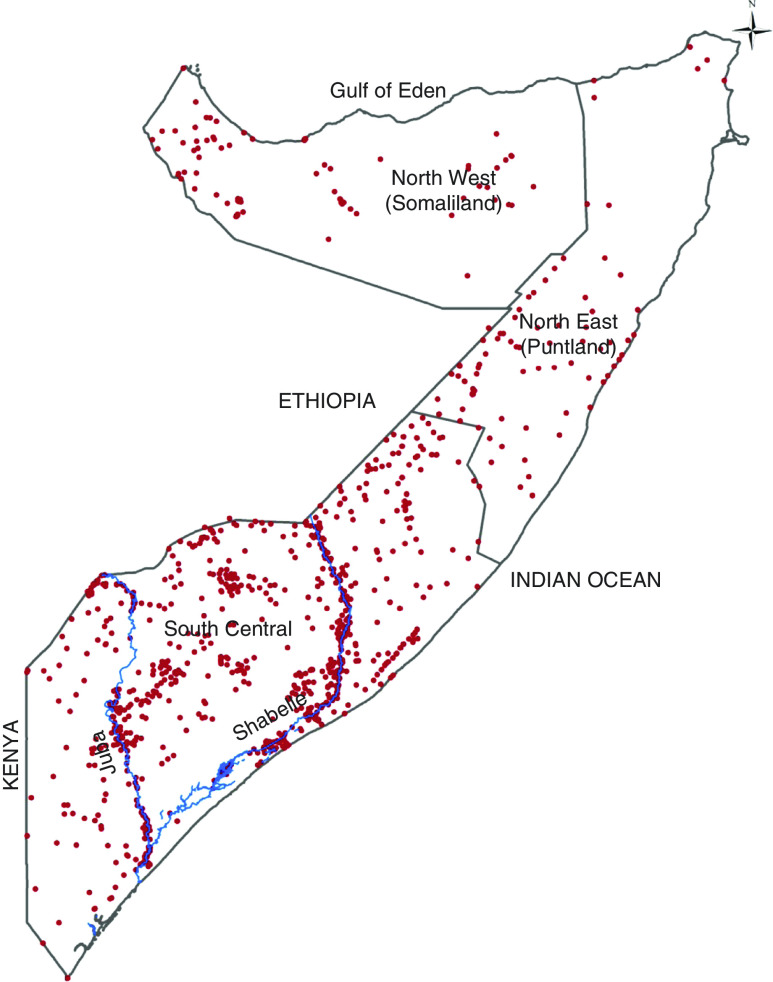
(colour online) Map showing the distribution of clusters sampled during the Food Security and Nutritional Analysis Unit nutrition surveys conducted between 2007 and 2010 in Somalia. The country is divided into three main zones: North West, North East and South Central

The FSNAU cross-sectional surveys were conducted biannually during the long (April to June) and short (October to November) rainy seasons between 2007 and 2010. A stratified, multistage cluster sampling design was used where the sampling frame of a selected district was based on three livelihood definitions (pastoral, agro-pastoral and riverine), within which thirty rural communities and thirty households within each community were selected at random^(^ ^20^ ^)^. Surveys were undertaken in all three zones of Somalia (Fig. 1).

Sample sizes for the surveys (number of households and number of children) were calculated by Standardized Methodology for Survey in Relief and Transition (SMART) methods^(^ ^9^ ^)^. A list of all villages and population within each of the assessed livelihoods was used to estimate the total population for the assessment area. The selection of households within the village was done randomly from a list of eligible names or a map of households where possible. Where these were not available, the number of households in the village was estimated from the population figures (the total population divided by the mean household size)^(^ ^21^ ^)^. Detailed descriptions of the survey methods and data collection are provided elsewhere^(^ ^9^ ^)^. The spatial coordinates for each cluster were derived from several spatial databases^(^ ^22^ ^)^.

Anthropometric measures were used to compute wasting and stunting using WHO 2006 references^(^ ^23^ ^)^. A child was defined as wasted or stunted when his/her *Z*-score for weight-for-age or height-for-age, respectively, was below −2. Additionally, children with MUAC below 125 mm were classified as having ‘low MUAC’. These measures were treated separately during analysis.

The predictors for the present study were selected using both the WHO conceptual framework on childhood stunting^(^ ^24^ ^)^ and the UNICEF conceptual framework of child health and survival^(^ ^18^ ^)^. The underlying predictors were related to household, maternal and environmental factors. At the child level, vitamin A supplementation in the last 6 months, diarrhoea, acute respiratory infection and incidence of febrile illness in the last 2 weeks before the survey, polio and measles vaccination history, sex and age of the child were examined in the present study. In addition, information was collected on child age, weight, height, MUAC and access to staple foods as well the mother’s age and MUAC. For each household, information recorded included the household size and age structure, sex of the household head and access to different types of foods in the last 24 h. Detailed description of the variables can be found in the online supplementary material (Table S1).

The effect of a set of five distal environmental covariates associated with vector-borne diseases^(^ ^25^ ^)^ and food security^(^ ^26^ ^)^ on the indices of malnutrition were examined. These were rainfall, enhanced vegetation index (EVI), mean temperature, distance to water features and urbanization. Rainfall and mean temperature were derived from the monthly average grid surfaces obtained from the WorldClim database^(^ ^27^ ^)^. The EVI values were derived from the MODerate-resolution Imaging Spectroradiometer (MODIS) sensor imagery^(^ ^28^ ^)^ for the period 2000–2010 while the urbanization information was obtained from the Global Rural Urban Mapping Project (GRUMP)^(^ ^29^ ^)^. All the environmental covariates were extracted from 1 km×1 km spatial resolution grids. Rainfall, temperature and EVI were summarized to compute seasonal averages using the four main seasons in Somalia: (i) December to March, the *Jilal* season, a harsh dry season; (ii) *Gu* which is the main rainy season from April to June; (iii) from July to September is the second dry season, the *Hagaa*; and (iv) the short rainy season known as *Deyr* from October to November. Further details of the covariates are provided in the online supplementary material (section S.1, ‘Data description’).

### Ethical approval

Ethical approval was provided through permission by the Ministry of Health Somalia, Transitional Federal Government of Somalia Republic (ref. MOH/WC/XA/146./07, dated 02/02/07). Informed verbal consent was sought from all participating households and individuals.

### Spatial-temporal Bernoulli regression model

Three separate Bayesian hierarchical spatial-temporal regression models were used to analyse the predictors of stunting, wasting and MUAC among children under the age of 5 years. Model parameters were estimated using the Integrated Nested Laplace Approximation (INLA) algorithm for inference and was implemented in R project version 3·0·1 using R-INLA library^(^ ^30^ ^)^.

Cluster-level effects were incorporated in the model to allow for the structured (spatial and temporal) and unstructured heterogeneity of malnutrition, using a convolution prior. District random effects were also included in this model. An assumption of additional flexibility in the model was made to allow for effects of non-linear predictors. Seasonality was controlled in the model as a factor with two unordered levels (April to June; October to November). A detailed description of the model procedures is provided in the online supplementary material (section S.2, ‘Spatial-temporal binomial regression model’).

## Results

Table 1 presents a summary of the survey data. Out of 1100 clusters sampled during the 2007–2010 nutritional surveys, 1066 clusters covering 73 778 children were included in the analysis. The longitude and latitude of 1765 children from thirty-four clusters could not be accurately determined and were excluded from the analysis. The mean household size was 6·0, with a mean number of children under 5 years of 2·0. The mean age of mothers was 31 years and in 81 % of households, the household head was a man. About half of children were reported to have received measles vaccination and 82 % had been inoculated against polio. Fever in the last 2 weeks was reported for 21 % of children, while 26 % and 17 % reported symptoms consistent with acute respiratory infection and diarrhoea, respectively. Over 90 % of children were in households which reported access to staple food sources of carbohydrates and proteins. However, only 41 % had access to fruits and vegetables. Sixty-three per cent of children lived in the mainly agricultural but conflict-prone South Central zone where 60 % of Somalia’s population resides; the rest were from the drier but more stable Somaliland and Puntland. By livelihood, 42 %, 27 % and 16 % of children were from areas of agro-pastoral, pastoral and riverine livelihoods, respectively, while 11 % and 4 % lived in internally displaced person camps and urban areas, respectively. The mean prevalence of wasting, stunting and MUAC<125 mm was 21 %, 31 % and 36 %, respectively. Distribution of weight-for-age, height-for-age and MUAC by year of survey, child age and sex can be found in Figs S1–S3 in the online supplementary material.

**Table 1:** Descriptive statistics of children under the age of 5 years in Somalia; data from household cross-sectional nutritional surveys conducted from 2007 to 2010

	*n*, mean or median	%, min–max or IQR
Sample size		
Total number of clusters examined	1066	–
Total number of children examined	73 778	–
Livelihood, *n* and %		
Agro-pastoral	30 844	42
Pastoral	20 004	27
Riverine	11 945	16
Urban areas	3237	4
Internally displaced persons	7748	11
Response variables, *n* and %		
Wasting	15 735	21
Stunting	22 739	31
MUAC <125 mm	26 425	36
Child data, *n* and %		
Vitamin A supplementation (yes)	41 739	57
Measles vaccination (yes)	37 908	51
Polio vaccination (yes)	60 844	82
Diarrhoea in the last 2 weeks (yes)	12 641	17
ARI in the last 2 weeks (yes)	18 939	26
Febrile illness in the last 2 weeks (yes)	15 675	21
Suspected measles in last 1 month (yes)	3942	5
Sex of the child=male	38 322	52
Age of the child (months), mean and min–max	33	6–59
Household data, median and IQR		
Household size	6	5–7
Number of under-5s	2	2–3
Age of the mother (years)	25	25–35
MUAC of the mother (cm)	25	23–25
Household head sex=male, *n* and %	60 128	81
Food and nutrition, *n* and %		
Carbohydrate in the last 24 h (yes)	71 517	97
Protein in the last 24 h (yes)	66 123	90
Fats in the last 24 h (yes)	58 062	79
Fruits and vegetables in the last 24 h (yes)	30 617	41
Climatic/environmental data		
Distance to major water features (km), *n* and %		
≤5 km	18 445	25
>5 km	55 333	75
EVI, mean and min–max	0·18	0–0·45
Precipitation (mm), mean and min–max	138	0–350
Temperature (°C), mean and min–max	28	21–31
Urbanization, *n* and %		
Urban	3318	5
Rural	70 460	95
Season, *n* and %		
April to June	47 327	64
October to November	26 451	36

IQR, interquartile range; MUAC, mid-upper arm circumference; ARI, acute respiratory infection; EVI, enhanced vegetation index.

The results from the spatial-temporal Bayesian logistic regression are shown in Table 2. The interaction effects between predictor variables were found to be very low and therefore not reported in these results. Among the child-level variables, fever and diarrhoea in the 2 weeks before the survey were significantly associated with wasting and stunting and low MUAC. Female children had a significantly lower likelihood of wasting and stunting, but a higher likelihood of low MUAC. Compared with children below the age of 12 months, older children had a lower prevalence of wasting and low MUAC, but a higher prevalence of stunting. Receiving measles vaccine or reporting a suspected episode of measles was not associated with any of the three measures of malnutrition. However, children who had received the polio vaccine had a lower likelihood of suffering from wasting.

**Table 2:** Spatial-temporal regression model outputs (OR and CrI) of the predictors of wasting, stunting and low MUAC in children under the age of 5 years in Somalia, 2007–2010

	Wasting		Stunting		MUAC<125 cm	
Predictors	OR	CrI	OR	CrI	OR	CrI
Child data						
Vitamin A supplementation	0·812	0·763, 0·865	0·998	0·947, 1·052	0·912	0·840, 0·991
Measles vaccination	1·024	0·961, 1·091	0·959	0·910, 1·011	0·989	0·910, 1·076
Polio vaccination	0·893	0·831, 0·961	1·089	1·023, 1·159	1·015	0·924, 1·115
Diarrhoea	1·349	1·272, 1·431	1·291	1·227, 1·359	1·792	1·666, 1·927
ARI	1·118	1·055, 1·186	1·013	0·964, 1·066	1·208	1·120, 1·303
Febrile illness	1·147	1·082, 1·217	1·156	1·098, 1·217	1·253	1·161, 1·352
Suspected measles	1·053	0·940, 1·180	1·055	0·955, 1·165	1·030	0·889, 1·194
Sex of the child (female)	0·727	0·693, 0·762	0·728	0·700, 0·758	1·237	1·162, 1·317
Age of the child (reference: <12 months)						
12–<24 months	0·809	0·739, 0·887	2·362	2·164, 2·579	0·776	0·707, 0·851
24–59 months	0·905	0·834, 0·982	2·021	1·862, 2·194	0·249	0·227, 0·273
Household data						
Household size	1·012	1·001, 1·023	1·024	1·014, 1·034	1·042	1·025, 1·059
Number of under-5s	1·085	1·054, 1·118	1·059	1·032, 1·087	1·059	1·016, 1·103
Female household head	0·960	0·901, 1·022	1·158	1·096, 1·223	0·899	0·824, 0·980
Age of the mother	1·002	0·998, 1·005	0·992	0·989, 0·995	1·001	0·997, 1·006
MUAC of the mother	0·989	0·985, 0·992	0·961	0·958, 0·964	0·983	0·978, 0·989
Food and nutrition						
Carbohydrate	0·834	0·792, 0·877	0·922	0·882, 0·965	0·983	0·919, 1·052
Protein	0·928	0·902, 0·955	0·916	0·894, 0·939	0·935	0·900, 0·971
Fats	1·056	0·989, 1·128	0·934	0·884, 0·988	0·961	0·883, 1·047
Fruits and vegetables	0·985	0·946, 1·025	1·064	1·029, 1·100	0·973	0·921, 1·027
Climatic/environmental data						
Season (reference: *Deyr*)	0·939	0·885, 0·996	0·984	0·933, 1·037	0·910	0·838, 0·989
Distance to water	1·000	1·000, 1·001	0·999	0·998, 1·000	0·998	0·997, 0·999
EVI	0·631	0·609, 0·655	0·498	0·482, 0·514	0·396	0·376, 0·417
Rainfall	0·999	0·998, 1·001	0·997	0·996, 0·998	0·982	0·980, 0·984
Temperature	1·084	1·043, 1·125	1·082	1·037, 1·129	1·107	1·031, 1·189
Urbanization	0·944	0·777, 1·147	1·180	0·989, 1·409	0·887	0·667, 1·179

CrI, credible interval; MUAC, mid-upper arm circumference; ARI, acute respiratory infection; EVI, enhanced vegetation index.

Prior vitamin A supplementation in the 6 months before the survey was associated with less wasting and low MUAC, but had no significant effect on stunting. Access to staple sources of proteins within the 24 h prior to the survey was associated with less malnutrition as defined by all three indicators. Children who had consumed any of the staple sources of carbohydrates within the 24 h prior to the survey had lower risk of wasting and stunting. Access to fruits and vegetables was associated with a small increase in stunting. Larger household size and number of children under 5 years were associated with small increases in malnutrition. Higher MUAC of the mother was also associated with a small reduction in all three categories of malnutrition.

Of the selected environmental covariates, the intensity of vegetation cover was the predictor that showed the greatest association with the three indicators of malnutrition. A 1-unit increase in EVI was associated with a 38 %, 49 % and 59 % reduction in wasting, stunting and MUAC<125 mm, respectively. Change of season from *Deyr* (October to November short rains) to *Gu* (April to June long rains) was associated with lower risk of wasting and underweight, but no association with stunting.

**Fig. 2:**
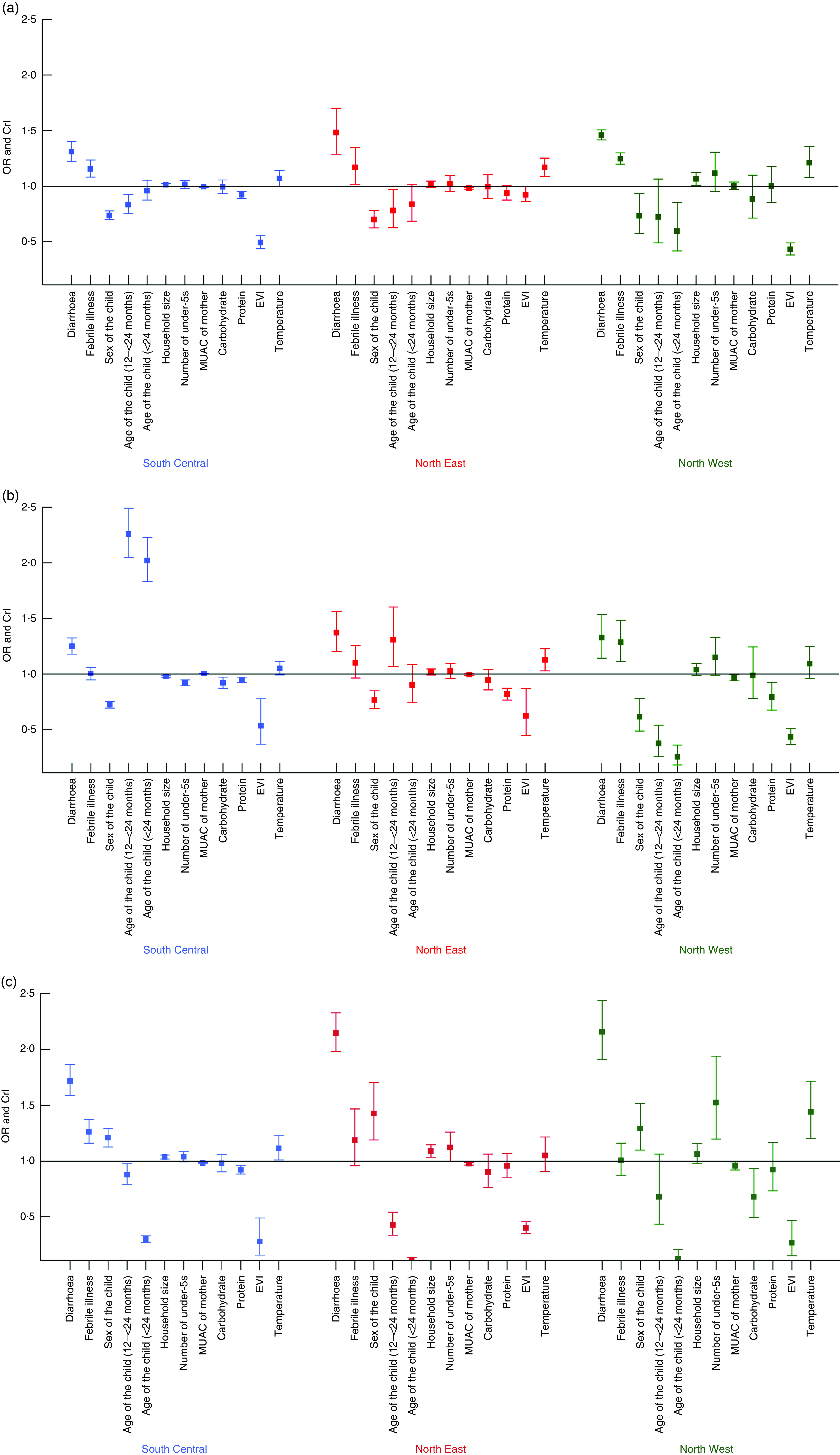
(colour online) Differences in the OR and CrI (represented by vertical bars) of the determinants of (a) wasting, (b) stunting and (c) low MUAC in children under the age of 5 years by zone in Somalia; data from household cross-sectional nutritional surveys conducted from 2007 to 2010 (CrI, credible interval; MUAC, mid-upper arm circumference; EVI, enhanced vegetation index)

Figure 2(a) to 2(c) shows the regional variation of important predictors associated with wasting, stunting and MUAC, respectively. Vegetation cover was found to be strongly associated with wasting, stunting and MUAC in the South Central and North West zones and to have a weak association in the North East zone, which is normally very dry and has large desert areas. Predictors with a strong association with wasting were diarrhoea and febrile illness in the last 2 weeks across all three regions. Stunting was associated with the age and sex of the child and environmental predictors related to vegetation cover and temperature, in all zones. Although the results show high variability in the effects of predictors associated with MUAC of the child, some of the predictors that showed a strong association were diarrhoea and febrile illness in the last 2 weeks, age of the child above 24 months and EVI.

## Discussion

In the present study, cross-sectional nutritional surveys undertaken from 2007 to 2010 were analysed to investigate the determinants of wasting, stunting and MUAC in Somalia. It represents the first nationwide formal analysis of the predictors of malnutrition in Somalia. The analysis shows that the average prevalence of wasting, stunting and MUAC<125 mm in Somalia from 2007 to 2010 was 21 %, 31 % and 36 %, respectively, values which meet the thresholds classified as ‘critical’ by the WHO^(^ ^18^ ^)^ (Table 1). The prevalence of malnutrition was highest in the Central South zone which has been the epicentre of conflict in Somalia in the last decade^(^ ^31^ ^)^.

Diarrhoea during the previous 2 weeks was associated with increases of approximately 35 % in wasting, 29 % in stunting and 20 % in the chance of having MUAC<125 mm^(^ ^14^ ^)^. The relationship of malnutrition and diarrhoea has been shown to be bidirectional. Diarrhoea may result in malnutrition because of reduced intake of nutrients, malabsorption, fecal nutrient losses and the effects of inflammatory response^(^ ^32^ ^)^. On the other hand, nutrient deficiency leads to loss of immunity which leads to frequent enteric infection among children^(^ ^12^ ^,^ ^15^ ^)^. A consistent association has been demonstrated between malnutrition and mortality from respiratory infections, where acute respiratory infection has been reported as a stronger predictor of malnutrition than diarrhoea^(^ ^33^ ^,^ ^34^ ^)^. Some studies show that acute respiratory infection is associated with higher risk of malnutrition in children due to a lack nutrients from breast-feeding which are crucial for child growth^(^ ^35^ ^)^.

Compared with children aged <12 months, older children (12–<24 months and 24–59 months) had a lower chance of wasting and MUAC<125 mm. However, these children were more than twice as likely to be stunted. Girls were 27 % less likely to be wasted or stunted but 24 % more likely to have MUAC<125 mm compared with boys below the age of 5 years. These results are consistent with findings in some low-income settings in Africa which show a higher prevalence of stunting in male than in female children^(^ ^34^ ^,^ ^35^ ^)^, but contrast with studies in Ethiopia^(^ ^17^ ^)^, Nigeria^(^ ^36^ ^)^ and Ghana^(^ ^11^ ^)^. Increase in the number of people in the household and in the number of children aged 6–59 months in a household were associated with high risk of malnutrition. This suggests that there is increased competition for household food in households with a high number of people^(^ ^37^ ^)^.

Interestingly, EVI, a proxy predictor of vegetation cover, was found to have the largest association with the three indices of malnutrition analysed in the present study. EVI is a satellite imagery-derived variable and characterizes the global range of vegetation state ranging from 0 (no vegetation cover) to 1 (high vegetation cover). Highly vegetated areas are a product of a combination of several variables including rainfall, seasonal and permanent water features, and to some extent underground water. In Somalia, it is an important correlate of availability of water for agriculture and pasture for livestock and may impact on household food security^(^ ^26^ ^)^. Given the large overall effect this index has on malnutrition and the susceptibility of Somalia to droughts, it is likely that its real relationship with malnutrition over the study period when a major drought occurred is underestimated.

Somalia, like most East and Horn of Africa countries, is prone to frequent droughts that often progress into famine, characterized by extreme food insecurity and malnutrition^(^ ^38^ ^)^. The large and significant associations of the malnutrition indicators in Somalia with the climatic indices such as EVI observed herein support this link. However, they also provide unique opportunities for using these climate indicators, which can be quantified at reasonable temporal and spatial resolutions^(^ ^28^ ^)^, to forecast possible risks of malnutrition to support effective planning. This approach can then be validated through rapid and inexpensive household surveys and may provide a dynamic process to support readiness to respond to emerging nutritional threats in the country.

Somalia is regarded as a country with some of the worst indicators of health in the world^(^ ^6^ ^)^ and in the current study the prevalences of fever and diarrhoea were relatively high and strongly associated with malnutrition. Regardless of the causal relationships, the majority of the available evidence on population health status in Somalia is likely to be suggestive and probably inadequate given the instability in the country and generally limited health research. Most available data are collected for programmatic purposes and are not necessarily hypothesis driven. Therefore, a stronger collaboration between non-governmental organizations and the regional research community should be fostered to help utilize existing programmatic infrastructure to collect health data through carefully designed studies that meet both operational and scientific needs. This will allow for a better analysis of the burden of ill health and risk factors in Somalia within the context of the ongoing instability.

There are some limitations to the present study. Information on access to water and sanitation, which contributes to the prevalence of diarrhoea, was not collected in the FSNAU surveys used herein. Household income was collected only in the 2007 and 2008 surveys and thus excluded from the set of predictors. The effect of the prolonged political conflict in Somalia was also not accounted for in the study. As shown in the results, the data come largely from rural areas and while urban areas were seen to have overall lower rates of undernutrition, there may be pockets of high levels of undernutrition not captured in the study. In addition, the location and timing of intervention programmes that may have affected the nutritional status of children and seasonal migration of the pastoral communities in search for pasture were not controlled for in our study.

## Conclusion

The present study represents the first formal nationwide analysis of the predictors of malnutrition in Somalia. It identifies some important distal predictors of wasting, stunting and MUAC among children in Somalia. Predictors shown to be important were related to household size, access to food and environmental indicators of vegetation and temperature. Major nutritional intervention programmes by international humanitarian organizations in Somalia focus mainly on improving nutritional status at the child level through targeted feeding programmes. Despite this, malnutrition rates in Somalia remain at critical levels. In the present study we have shown that there are other important underlying factors at household and community levels that are associated with malnutrition in children and that require a wider response. Timely and targeted interventions at different seasons to mitigate the effects of rainfall variability, and hence vegetation and agriculture, should be investigated.

## Supplementary material

For supplementary material accompanying this paper visit https://doi.org/10.1017/S1368980015001913. click here to view supplementary material
