# Supplementary material for: Predictors of the risk of malnutrition among children under the age of 5 years in Somalia
Source: Public Health Nutr. 2015 Jun 16;18(17):3125–33. doi: 10.1017/S1368980015001913 (PMC4697134; doi:10.1017/S1368980015001913)
Supplement: Supplementary file 1 [file S1368980015001913sup001.docx]

**Supplementary material**

**Data description, model procedures and model output**

**S.1 Data description**

The anthropometric indicators used as response variables were: weight-for-height (wasting); height-for-age (stunting) and mid upper arm circumference (MUAC)-for-age. The cut-off point of < -2SD used for wasting and stunting was derived from WHO 2006 reference (1). The predictor variables were grouped into three categories; child level characteristics, household characteristics which also covers the access to food and nutrition in the household; and environmental and climatic predictors. The anthropometric indices, child and household level covariates were obtained from surveys conducted bi-annually between 2007 and 2010 by the Food Security and Nutrition Analysis Unit (FSNAU) of the Food and Agriculture Organization of United Nations (UNFAO).

A set of five geographical covariates were examined; rainfall, enhanced vegetation index (EVI), temperature, distance to water and urbanization. Rainfall and temperature were derived from the monthly average rasters obtained from WorldClim datasets and were summarized to get the seasonal mean rainfall and annual mean temperature (2). The EVI values were derived from the MODerate-resolution Imaging Spectroradiometer (MODIS) sensor imagery (3) while the urbanization was obtained from Global Rural Urban Mapping Project (GRUMP) (4). Table SI.1 gives a summary description of every variable used in this study. Figure SI.1 and SI.2 are snapshot display of distribution of the response variables by year of survey, age and sex of the child.

In Somalia, the livelihoods are broadly based on subsistence farming and pastoralism with limited opportunities to earn wages. Therefore livelihoods zones in Somalia are mainly: agro-pastoral, pastoral and riverine. Communities were defined as pastoral if they engaged primarily in livestock production and were nomadic (moved with their livestock from place to place in search of water and pasture); agro-pastoral if they practiced mixed crop and livestock production; and riverine if they lived along the river and were primarily involved in crop production and river-based economy (5).

The season of survey was as well derived from the FSNAU surveys depending the month of survey. Somalia has four main seasons around which pastoral and agricultural activities depend: December to March is the ‘Jilal’ season, a harsh dry season; ‘Gu’ which is the main rainy season from April to June; from July to September is the second dry season, the ‘Hagaa’; and the short rainy season known as ‘Deyr’ from October to December (6). Seasonality was also controlled in the model using two unordered level to reflect the time of surveys (Gu and Deyr).

**Table S1:** This table gives detailed information of the variables that were used in the study. Three response variables were defined namely; wasting, stunting and MUAC-for-age using WHO standard population and cut-off points. The predictors were divided into three categories; child specific predictors, household level predictors which also included food and nutrition predictors, and climatic or environmental variables.

| **Variable** | **Type** | **Description** |
| --- | --- | --- |
| **Response variables** | | |
| Wasting | Categorical | 1 = wasted (cut-off point <-2 SD = malnutrition)  0 = otherwise (cut-off point <-2 SD = malnutrition) |
| Stunting | Categorical | 1 = stunted (cut-off point <-2 SD = malnutrition)  0 = otherwise |
| (MUAC)-for-age | Categorical | 1 = malnourished (cut-off point <-125mm = malnutrition)  0 = otherwise (cut-off point <-2 SD = malnutrition) |
| **Child specific predictors** | | |
| *Pf* Malaria positive | Categorical | 1 = malaria positive (RDT positivity)  0 = otherwise |
| Vitamin supplements | Categorical | 1 = yes (In the last 6 months)  0 = otherwise |
| Measles vaccinations | Categorical | 1 = yes  0 = otherwise |
| Polio vaccination | Categorical | 1 = yes (Complete doses)  0 = otherwise |
| Diarrhoea | Categorical | 1 = diarrhoea (In the last 2 weeks)  0 = otherwise |
| Acute Respiratory Infection (ARI) | Categorical | 1 = ARI positive (In the last 2 weeks)  0 = otherwise |
| Febrile Illness | Categorical | 1 = fever positive (in the last 2 weeks)  0 = otherwise |
| Suspected measles | Categorical | 1 = yes (In last 1 month)  0 = otherwise |
| Gender | Categorical | 1 = Female  0 = otherwise |
| Age of the child | Continuous | Age of children from 6 to 59 months (in months) |
| **Household level predictors** | | |
| Household size | Continuous | Number of people in the household |
| Number of under fives | Continuous | Number of children under the age of five years in the household (0-59 months) |
| Household gender female | Categorical | 1= Female  0 = Male |
| Age of the mother | Continuous | Age of the mother in years |
| MUAC of the mother | Continuous | MUAC of the mother in cm |
| **Food and nutrition predictors** | | |
| Carbohydrates | Categorical | 1 = yes (Access to at least one type of carbohydrates in the last 24 hours)  0 = otherwise |
| Protein | Categorical | 1 = yes (Access to at least one type of protein in the last 24 hours)  0 = otherwise |
| Fats | Categorical | 1 = yes (Access to at least one type of Fat in the last 24 hours)  0 = otherwise |
| Fruits and vegetables | Categorical | 1 = yes (Access to at least one type of Fruits and vegetables in the last 24 hours)  0 = otherwise |
| **Climatic / Environmental data** |  |  |
| Distance to water | Continuous | In km, user derived using ArcGIS 10.1 (ESRI Inc. NY, USA) |
| Enhanced Vegetation Index (EVI) | Continuous | Ranges from 0-1 and derived from temporal Fourier analysed Advanced Very High Resolution Radiometer (AVHRR) data |
| Rainfall | Continuous | Seasonal mean rainfall in mm obtained from WorldClim dataset |
| Temperature | Continuous | Annual mean temperature in ˚C obtained from WorldClim dataset |
| Urbanization | Categorical | Global Rural Urban Mapping Project Modified (GRUMPMod), 1=Urban, 0=Rural |
| Season | Categorical | 1=Gu (April to June), 2=Deyr (October to November) |


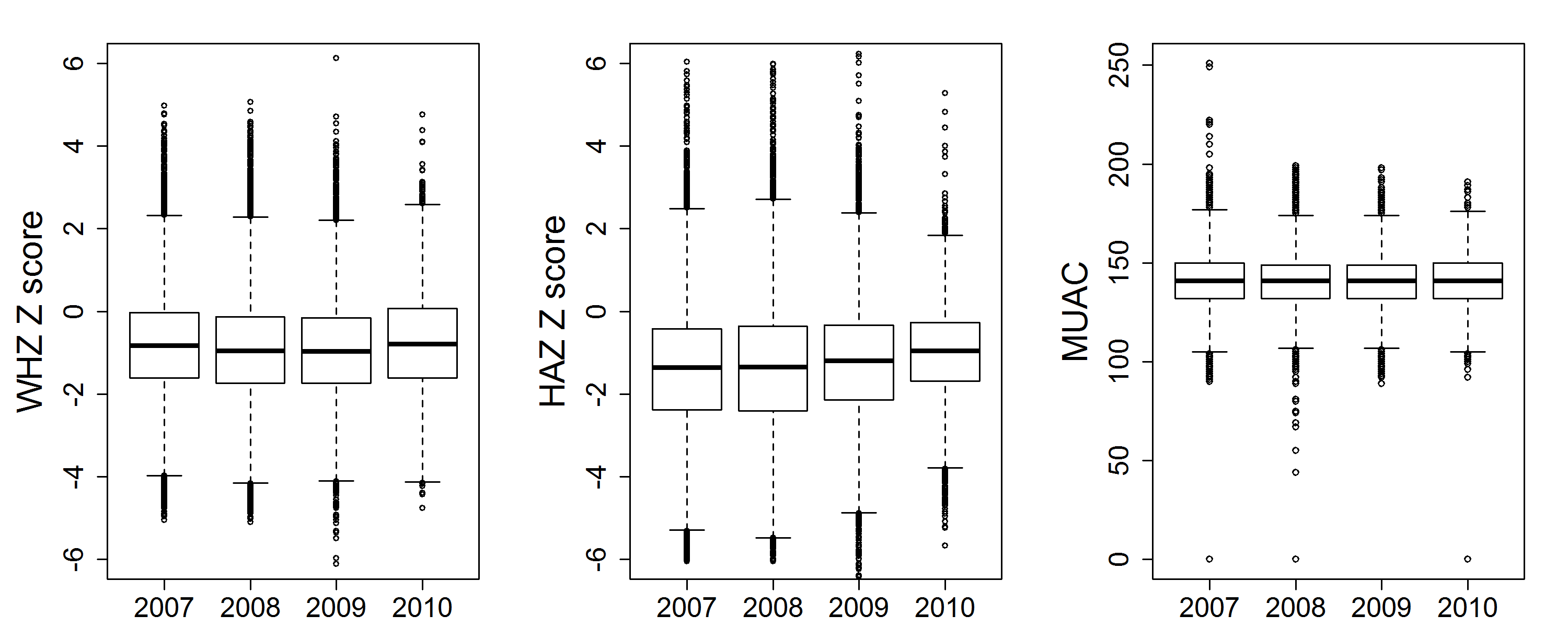

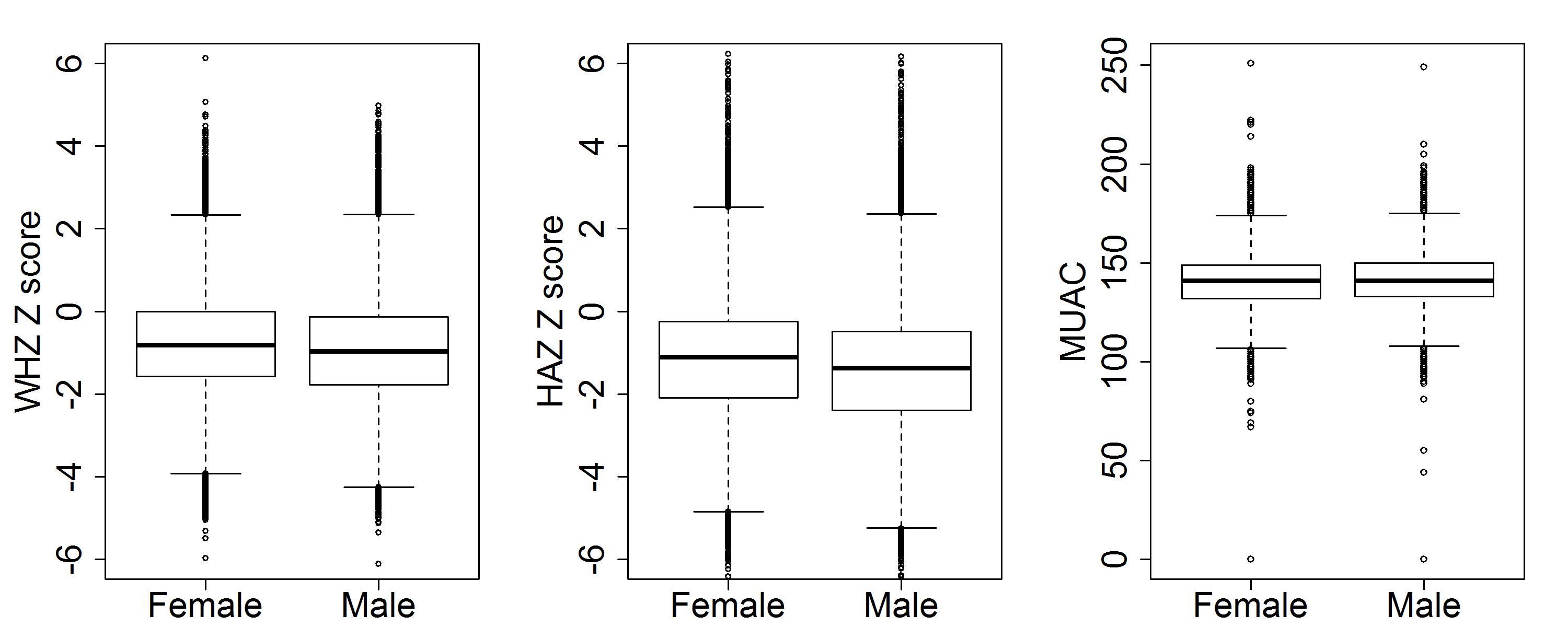


**Figure S1:** Box plots showing the distribution of the weight-for-height, height-for-age, and mid upper arm circumference (MUAC) by year of survey and gender of the child. WHZ Z score= weight-for-height Z score, HAZ Z score = height-for-age Z score, MUAC= Mid Upper Arm Circumference of the child.


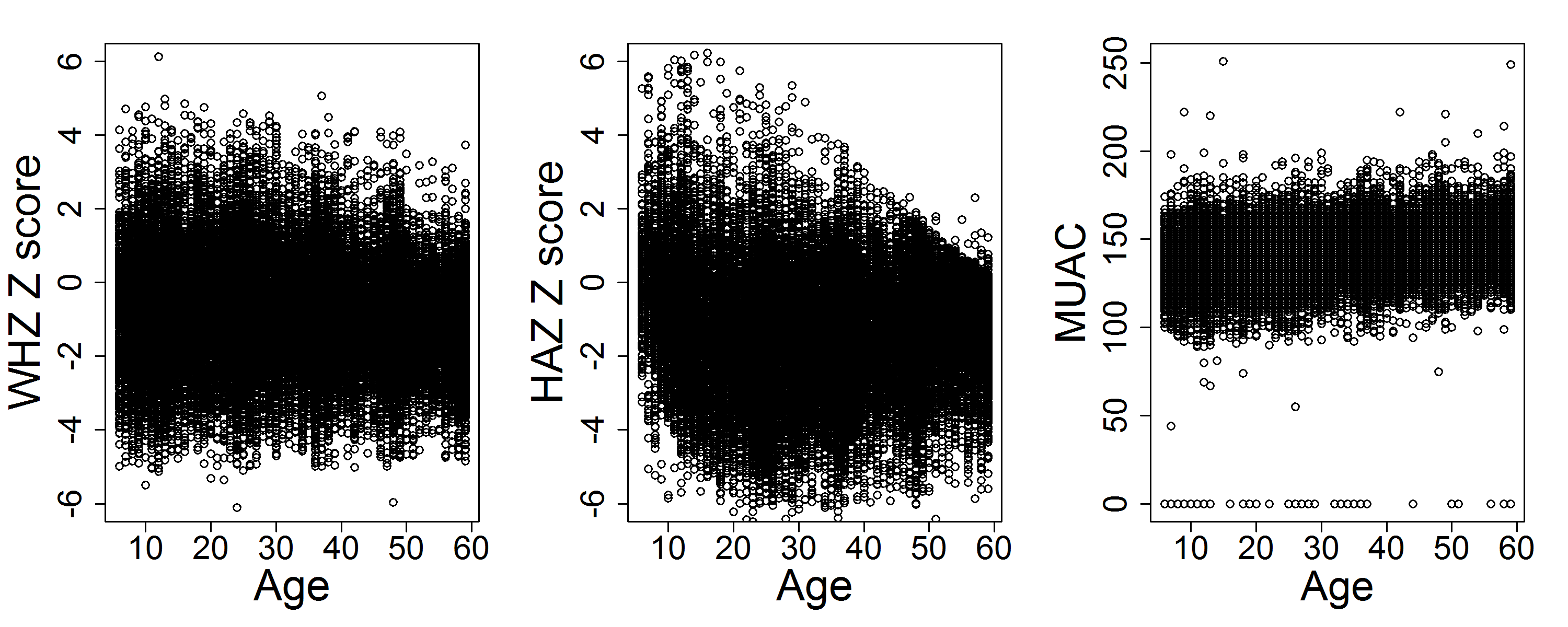

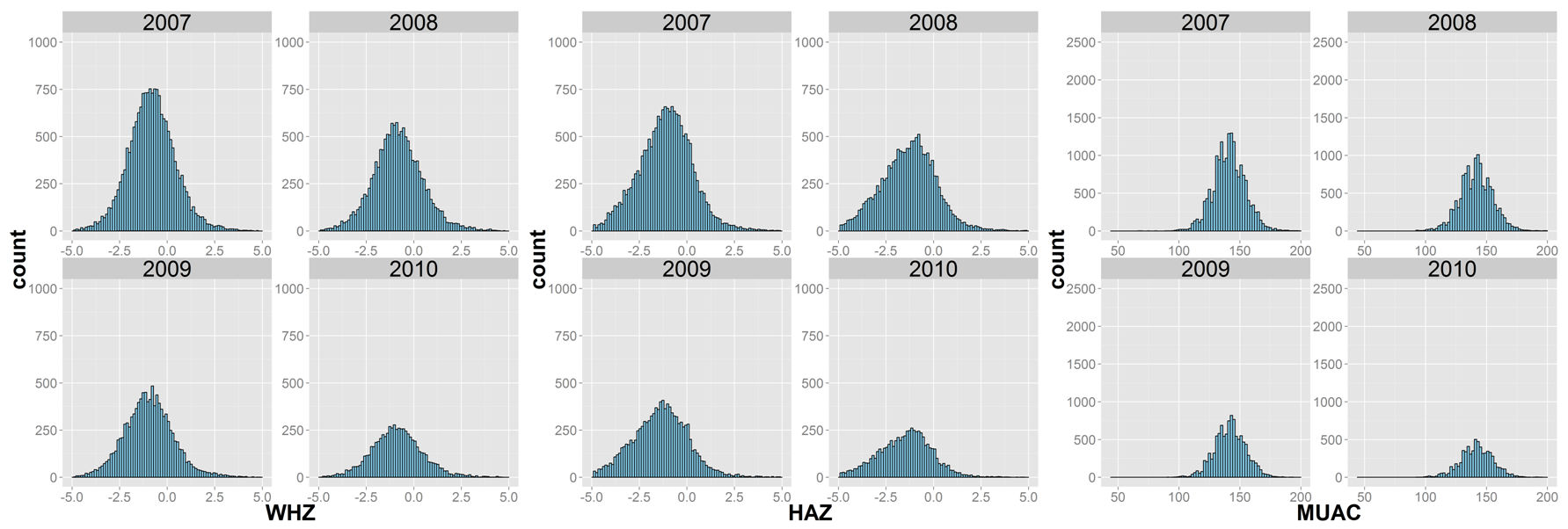


**Figure S2:** Scatter plot and histograms displaying the distribution of the three indicators of malnutrition by age of the child in months and years of survey. WHZ_WHO= weight-for-height Z score, HAZ_WHO = height-for-age Z score, MUAC= Mid Upper Arm Circumference of the child.


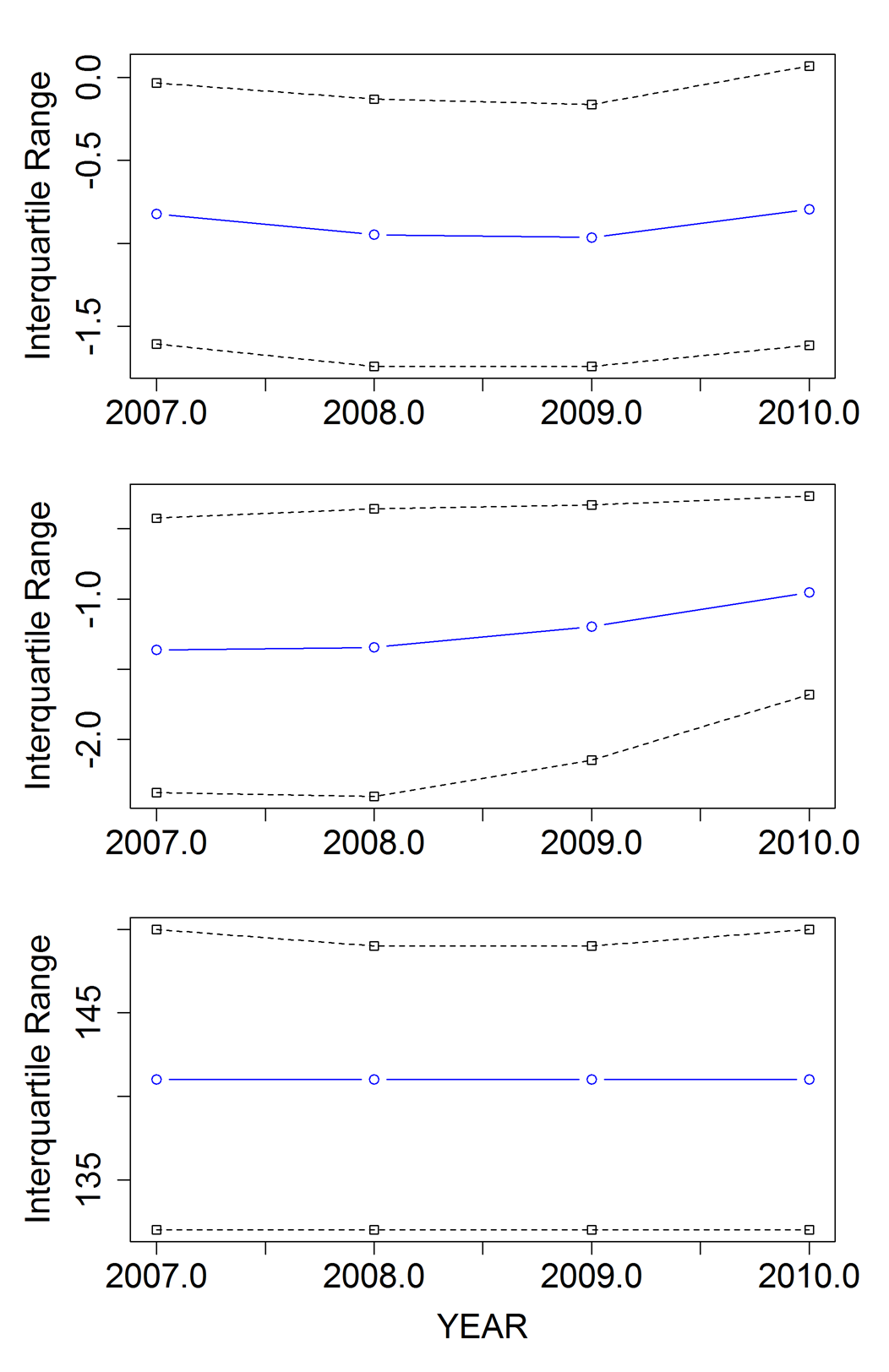


**Figure S3:** Plots of median and inter-quintile range of the three indicators of malnutrition by years of survey. WHZ_WHO= weight-for-height Z score, HAZ_WHO = height-for-age Z score, MUAC= Mid Upper Arm Circumference of the child.

#### S.2 Spatial-temporal binomial regression model

Specifically, we let $y_{i}$ to be a binary response variable where $y_{i}=1$ if a child is malnourshed and $y_{i}=0$ if a child is not malnourished where $i;i=1,\ldots,73778.$ We assumed that this number was Binomial with a cluster level prevalence $p_{i}$ , the response variable is distributed as a Bernoulli random variable such that

$f(\mathrm{y}_{i}|\mathrm{n}_{i})=\mathrm{p}_{i}^{y_{i}}(1-p_{i})^{1-y_{i}}=\exp[\mathrm{y}_{i}\mathrm{n}_{i}-\log(1+\exp(\mathrm{n}_{i}))]$ (Equation SI2.1)

where $p_{i}=P(\mathrm{y}_{i}=1),$ and $n_{i}=\log it(\mathrm{p}_{i})$ is a canonical parameter linked to the linear predictor. We modeled the relationship between malnutrition and predictors using a Bayesian hierarichical logistic regression model that account for excess heterogeneity and spatial similarity between clusters.

This model is expressed as

$\log\text{it}\left(p_{i}\right)=X_{i}^{'}\beta+u_{i}+v_{i}$, (Equation SI2.2)

where $\beta$ is a $\left(p\times1\right)$vector of parameters, $X_{i}^{'}$is the covariate matrix, $u_{i}$is a spatial structured random effect for cluster$i$, $v_{i}$ is an unstructured random effect for cluster$i$.

To model spatial dependence between the random effects $u_{i}$ in neighbouring areas we assumed a conditional autoregressive (CAR) prior for these terms. Each $u_{i}$ was modelled (conditional on its weighted neighbors) as normally distributed with mean equal to the mean of the effects of its neighbors and a variance that is inversely proportional to the number of neighbours $N_{i}$, that is, $u_{i}|u_{-i}\sim N\left(\mu_{i},\sigma_{i}^{2}\right)$, where $u_{-i}$ is a vector of all the spatial random effects except for the one at the i-th cluster and and , where $n_{i}$ is the number of clusters in the neihbourhood of the i-th cluster, and $\tau$ is the precision parameter. The joint distribution of the$v_{i}$’s is an independent multivariate normal.

**References**

x

| 1. | de Onis M, others. WHO Child Growth Standards based on length/height, weight and age. Acta Paediatrica. 2006; 95(Supplement 450): p. 76-85. |
| --- | --- |
| 2. | Murtaugh PA. Performance of several variable-selection methods applied to real ecological data. Ecology Letters. 2009; 12(10): p. 1061-1068. |
| 3. | Scharlemann JP, Benz D, Hay SI, Purse BV, Tatem AJ, Wint GW, et al. Global data for ecology and epidemiology: a novel algorithm for temporal Fourier processing MODIS data. PLoS One. 2008; 3(1): p. e1408. |
| 4. | Schneider A, Friedl M, Potere D. A new map of global urban extent from MODIS satellite data. Environmental Research Letters. 2009; 4(4): p. 044003. |
| 5. | Noor AM, Moloney G, Borle M, Fegan GW, Shewchuk T, Snow RW. The use of mosquito nets and the prevalence of Plasmodium falciparum infection in rural South Central Somalia. PLoS One. 2008; 3(5): p. e2081. |
| 6. | Hadden RL. The Geology of Somalia: A Selected Bibliography of Somalian Geology, Geography and Earth Science. Tech. rep. DTIC Document; 2007. |

x
